# Adaptation of Hepatitis C Virus to Mouse CD81 Permits Infection of Mouse Cells in the Absence of Human Entry Factors

**DOI:** 10.1371/journal.ppat.1000978

**Published:** 2010-07-01

**Authors:** Julia Bitzegeio, Dorothea Bankwitz, Kathrin Hueging, Sibylle Haid, Christiane Brohm, Mirjam B. Zeisel, Eva Herrmann, Marcus Iken, Michael Ott, Thomas F. Baumert, Thomas Pietschmann

**Affiliations:** 1 Division of Experimental Virology, TWINCORE, Centre for Experimental and Clinical Infection Research; a joint venture between the Medical School Hannover (MHH) and the Helmholtz Centre for Infection Research (HZI), Hannover, Germany; 2 Inserm, U748, Université de Strasbourg, Strasbourg, France; 3 Institute for Biostatistics and Mathematical Modeling, Johann Wolfgang Goethe-University, Frankfurt, Germany; 4 Department of Gastroenterology, Hepatology and Endocrinology, Hannover Medical School, Germany, and Clinical Research Group Cell and Gene Therapy, TWINCORE, Centre for Experimental and Clinical Infection Research, Hannover, Germany; Washington University School of Medicine, United States of America

## Abstract

Hepatitis C virus (HCV) naturally infects only humans and chimpanzees. The determinants responsible for this narrow species tropism are not well defined. Virus cell entry involves human scavenger receptor class B type I (SR-BI), CD81, claudin-1 and occludin. Among these, at least CD81 and occludin are utilized in a highly species-specific fashion, thus contributing to the narrow host range of HCV. We adapted HCV to mouse CD81 and identified three envelope glycoprotein mutations which together enhance infection of cells with mouse or other rodent receptors approximately 100-fold. These mutations enhanced interaction with human CD81 and increased exposure of the binding site for CD81 on the surface of virus particles. These changes were accompanied by augmented susceptibility of adapted HCV to neutralization by E2-specific antibodies indicative of major conformational changes of virus-resident E1/E2-complexes. Neutralization with CD81, SR-BI- and claudin-1-specific antibodies and knock down of occludin expression by siRNAs indicate that the adapted virus remains dependent on these host factors but apparently utilizes CD81, SR-BI and occludin with increased efficiency. Importantly, adapted E1/E2 complexes mediate HCV cell entry into mouse cells in the absence of human entry factors. These results further our knowledge of HCV receptor interactions and indicate that three glycoprotein mutations are sufficient to overcome the species-specific restriction of HCV cell entry into mouse cells. Moreover, these findings should contribute to the development of an immunocompetent small animal model fully permissive to HCV.

## Author Summary

The hepatitis C virus (HCV) infects only humans and chimpanzees, which has hampered development of suitable animal models. The inability of HCV to penetrate non-human cells is primarily due to inefficient usage of non-human CD81 and occludin. In this study we adapted HCV to mouse CD81. Efficient utilization of mouse CD81 is conferred by a combination of three mutations in the viral glycoproteins. These changes also permit entry via rat or hamster CD81, and lower viral dependence on additional HCV entry factors. Strikingly, mouse CD81 adapted HCV glycoproteins mediate entry into mouse cells in the absence of human entry factors. The adaptive mutations are not resident in viral domains implicated in direct CD81 binding. Nevertheless, they enhance binding to human CD81, increase susceptibility to different neutralizing antibodies and facilitate induction of viral cell fusion by low pH. This suggests that structural changes accompanied by exposure of the CD81 binding site and neutralizing epitopes have “unlocked” the viral envelope protein complex facilitating infection through non-human entry factors. These results highlight mechanisms of HCV receptor usage and tropism. They also demonstrate that HCV can be adapted to using non-human host factors, which may ultimately facilitate the development of small animal models.

## Introduction

HCV is an enveloped virus with a positive sense single stranded RNA genome, belonging to the family of *Flaviviridae* [1]. Based on sequence comparison patient isolates are classified into seven genotypes which differ from each other by ca. 31–33% at the nucleotide level [2], [3]. Chronic hepatitis C is associated with severe liver disease including hepatitis, liver cirrhosis and hepatocellular carcinoma and it is the most frequent indication for liver transplantation [4]. Presently, neither therapeutic nor preventive vaccines are available and the only current therapy, a combination of pegylated interferon-α and ribavirin, is limited by resistance, toxicity and high costs [5].

Development of HCV-specific antivirals and vaccines has been impeded by lack of convenient animal models. Besides humans the only host for HCV is the chimpanzee and an immunocompetent small animal model is still lacking. The restricted tropism of HCV likely reflects specific host factor requirements for entry, RNA-replication, assembly and release of virions. Although mouse cells have been shown to sustain HCV RNA replication [6], the efficiency is low and release of progeny virus particles was not observed [7]. Moreover, cell entry of HCV into rodent cells is possible, but requires overexpression of essential human entry factors [8]. Therefore, propagation of HCV in mouse cells is likely restricted at multiple steps of the viral replication cycle.

HCV cell entry is a complex process involving a number of host factors. Initial adsorption of viral particles consisting of core protein, envelope proteins 1 and 2 (E1, E2) and associated with lipoproteins [9]–[11], may be facilitated by attachment factors like glycosaminoglycans [12], the LDL receptor [13], [14] and lectins [15]–[17]. Beyond these, four essential HCV entry factors have been identified: the scavenger receptor class B type 1 (SR-BI) the tetraspanin CD81 and the tight junction proteins claudin-1 (CLDN1) and occludin (OCLN) [8], [18]–[22]. Usage of these four factors mediates clathrin-dependent endocytosis [23] of virus particles and ultimately permits viral membrane fusion in a low pH-triggered fashion [24]. Notably, ectopic expression of human SR-BI, CD81, CLDN1 and OCLN rendered all cell lines tested permissive for HCV suggesting that additional host factors necessary for host cell entry are broadly expressed and conserved among different mammalian species [8]. Meanwhile, accumulating evidence indicates that a species-specific interplay of HCV with these four entry factors restricts HCV entry into non-human cells and thus contributes to HCV's narrow species tropism [25]–[27]. Particularly, inefficient usage of non-human CD81 and OCLN by HCV seems to limit viral entry into non-human cells [8]. However, more recently evidence was provided that mouse SR-BI and at least for genotype 2a also mouse CLDN1 supported HCV infection with slightly lower efficiency compared to their human homologs [26], [27], implying that incompatibility of these factors may also limit HCV entry into mouse cells.

The interaction between the viral glycoprotein E2 and CD81 has been analyzed using truncated, soluble E2 and retroviral pseudoparticles bearing the HCV glycoproteins E1 and E2 (HCVpp). These studies highlight that defined residues within the large extracellular loop (LEL) of CD81, a protein domain that is highly variable between CD81 isoforms from different species, are crucial for interaction with HCV E2 [28], [29]. On the other hand, the viral binding site for CD81 seems to be a conformational epitope within E2: Based on reverse genetics and antibody competition experiments with E2-specific antibodies, multiple discontinuous epitopes have been implicated in CD81 binding [30]–[34].

To characterize viral determinants for CD81 usage and to assess if improved utilization of mouse CD81 is sufficient to permit entry of HCV into mouse cells, we adapted HCV to mouse CD81, and analyzed the consequences for virus receptor interaction and tropism.

## Results

### Adaptation of HCV to mouse CD81

**Figure 1:**
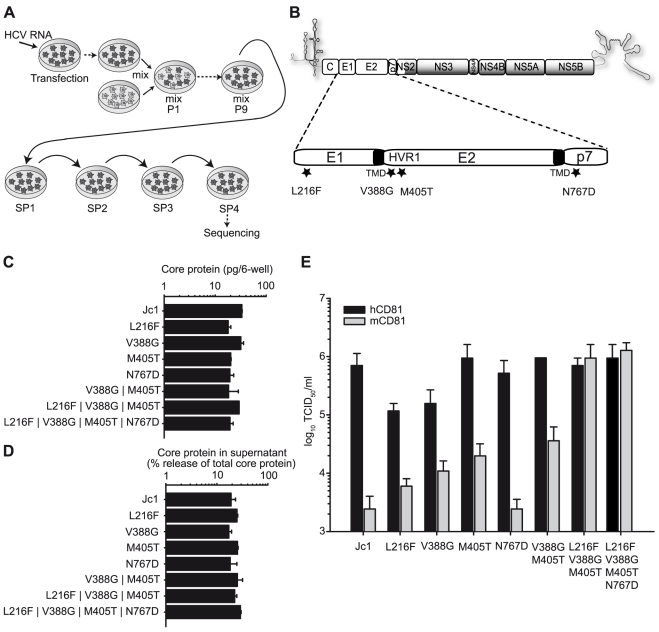
Adaptation of Jc1 to mouse CD81. (A) Schematic overview of the process of adaptation. Lunet N mCD81 cells were transfected with Jc1 RNA by electroporation (arrow), passaged 5 times and then mixed with untransfected Lunet N mCD81 cells (mix). The cells were continuously passaged until the virus had spread to the whole cell population (mix P1–P9) followed by four consecutive supernatant passages (SP). RNA from the fourth SP was extracted, cloned and sequenced. (B) Jc1 genome organization. The viral glycoproteins E1 and E2 and the p7 ion channel protein are enlarged. The mutations identified by sequencing of the passaged virus population are indicated with stars. J6CF-derived proteins segments are depicted by white boxes, JFH1-derived proteins are shaded in grey. Transmembrane domains (TMD) of E1 and E2 are depicted in black. HVR1, hypervariable region 1. (C+D) Huh-7.5 cells were transfected with Jc1 and indicated Jc1-based mutant RNAs. The amount of intracellular core protein in cell lysates (C) and the percent of total core protein production released into the extracellular space was determined 48 h post transfection by a core-specific ELISA (D). Mean values of duplicate measurements and the standard deviation of the means are given. (E) Infectivity of Jc1 and indicated Jc1 derivatives was determined on Lunet N cells expressing either human or mouse CD81 using the limiting dilution assay (TCID_50_). Prior to titration virus preparations were normalized for equal quantity of core protein.

For adaptation of HCV to mouse CD81, we first created Huh7-Lunet cells [35] that are highly permissive for HCV RNA replication and virus production but that are essentially not infectable due to limiting expression of endogenous human CD81. This cell clone designated Lunet N [36] was created by fluorescence activated cell sorting for cells expressing very little CD81. The resulting cell population was subcloned and individual clones were analyzed for CD81 expression and permissiveness for HCV RNA replication. Ultimately, a subclone with minimal residual CD81 expression supporting high level HCV RNA replication was selected. In the absence of ectopically expressed CD81, these cells designated Lunet N cells are essentially refractory to HCV infection (Figure S1). Susceptibility of these cells to HCV infection was restored by ectopic expression of human or mouse CD81. However, in agreement with the results of Flint et al. [25], mouse CD81 supported infection approximately 100-fold less efficiently than human CD81 (Figure S1). Next, we transfected Lunet cells expressing mouse CD81 (Lunet N mCD81) with the HCV genotype 2a chimeric virus Jc1 that grows to high virus titers [37]. After five cell passages, naive Lunet N mCD81 cells were added to the culture and nine additional cell passages later, virus had spread to nearly all cells of the co-culture (data not shown). At this time point, four consecutive passages of cell-free culture fluid to naïve Lunet N mCD81 cells were conducted to select for virus variants that efficiently use mouse CD81 (Figure 1A). At individual passage steps of the procedure, we sampled the infectivity of the virus population present on cells expressing either human, mouse or no CD81 to monitor the progress of adaptation.

In the course of this experiment, viruses were selected that remained dependent on CD81 but which were able to use mouse and human CD81 with comparable efficiency (Figure S2). HCV RNA derived from the final supernatant passage was isolated and sequenced. In the region of the genome encoding the viral structural proteins Core, E1, E2 as well as p7, NS2 and NS3, we identified four mutations that were conserved at least among 4 out of the 6 sequenced clones (Figure 1B). Three of these mutations were located in the viral glycoproteins; one in E1 (L216F) and two in E2 (V388G and M405T). Both mutations located in E2 reside within the hypervariable region 1 (HVR1), a stretch of 27 amino acids at the N-terminus of E2 which has been implicated to interact with SR-BI [38]. The fourth mutation was detected in the first transmembrane domain of the p7 protein (N767D). To analyze the influence of these amino acid changes on RNA-replication, virus production and infection of cells expressing human or mouse CD81, we transferred them individually or in combinations into the backbone of Jc1. These constructs were then transfected into naïve Huh-7.5 cells, and intracellular as well as extracellular core protein levels were determined to assess replication and virus release (Figure 1C–1D). Finally, cell-free culture fluids were harvested, normalized for equal quantity of core and then used to infect Lunet N cells expressing either human or mouse CD81 (Figure 1E). None of the mutations individually or in combination markedly affected RNA-replication and virus release, as is evident from comparable levels of intracellular core 48 h post transfection of these constructs and from a similar efficiency of core protein release at this time point (Figure 1C–1D). The mutation in p7 did not affect virus entry into cells with mouse or human CD81 and thus CD81 usage. In contrast, all three mutations in the glycoproteins moderately increased the efficiency of the virus to infect cells carrying mouse CD81 compared to the parental Jc1 chimera (Figure 1E) thus suggesting that these changes enhance the ability of the virus to use mouse CD81. Interestingly, L216F and V388G at the same time reduced efficiency of infection via the human CD81 counterpart. In contrast, the M405T mutation stimulated infection through mouse CD81 without affecting entry via human CD81. Strikingly, the combination of all three glycoprotein-resident mutations as well as all four mutations together, increased infection mediated by mouse CD81 to the levels sustained by human CD81 (Figure 1E). Notably, infectivity of Jc1 carrying a combination of the L216F, V388G and M405T mutations (designated Jc1/mCD81) on Lunet N mCD81 cells was efficiently neutralized by antibodies specific to mouse CD81 but not by antibodies directed against human CD81, thus indicating that the virus indeed uses the mouse entry factor for infection (data not shown).

**Figure 2:**
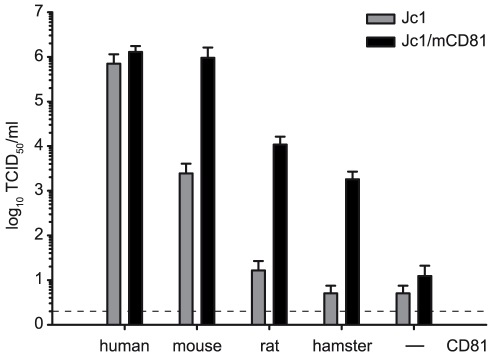
Adaptive mutations increase HCV cell entry via different rodent CD81 molecules. Jc1 and Jc1/mCD81 stocks were generated by transfection of Huh-7.5 cells. Cell supernatants were collected 48 h after tansfection and titrated on the indicated cell lines using the limiting dilution assay. The dashed line indicates the detection limit of the limiting dilution assay.

To address if these mutations specifically permit infection via mouse CD81 or if they would also increase HCV infection through CD81 from other rodent species, we cloned rat and hamster CD81 and stably transduced Lunet N cells with these orthologs. These cells were challenged with equal quantities of Jc1 and Jc1/mCD81 particles. Similar to the mouse ortholog, infection through rat and hamster CD81 was approximately 100-fold more efficient with Jc1/mCD81 compared to parental Jc1 (Figure 2). Thus, these three mutations within HCV E1 and E2 enhance entry efficiency of HCV through mouse, rat and hamster CD81 to a comparable degree suggesting that the adaptation was not specific to mouse CD81 only. It is worth mentioning here that both parental Jc1 and Jc1/mCD81 infected Lunet N cells, albeit with very low efficiency (Figure 2). Since this low level infectivity was susceptible to neutralization by CD81-specific antibodies it is likely due to very low residual levels of human CD81 in these cells rather than due to a CD81-independent route of virus entry (data not shown).

### Adaptation enhances HCV interaction with soluble human CD81 and increases exposure of the viral CD81 binding site

**Figure 3:**
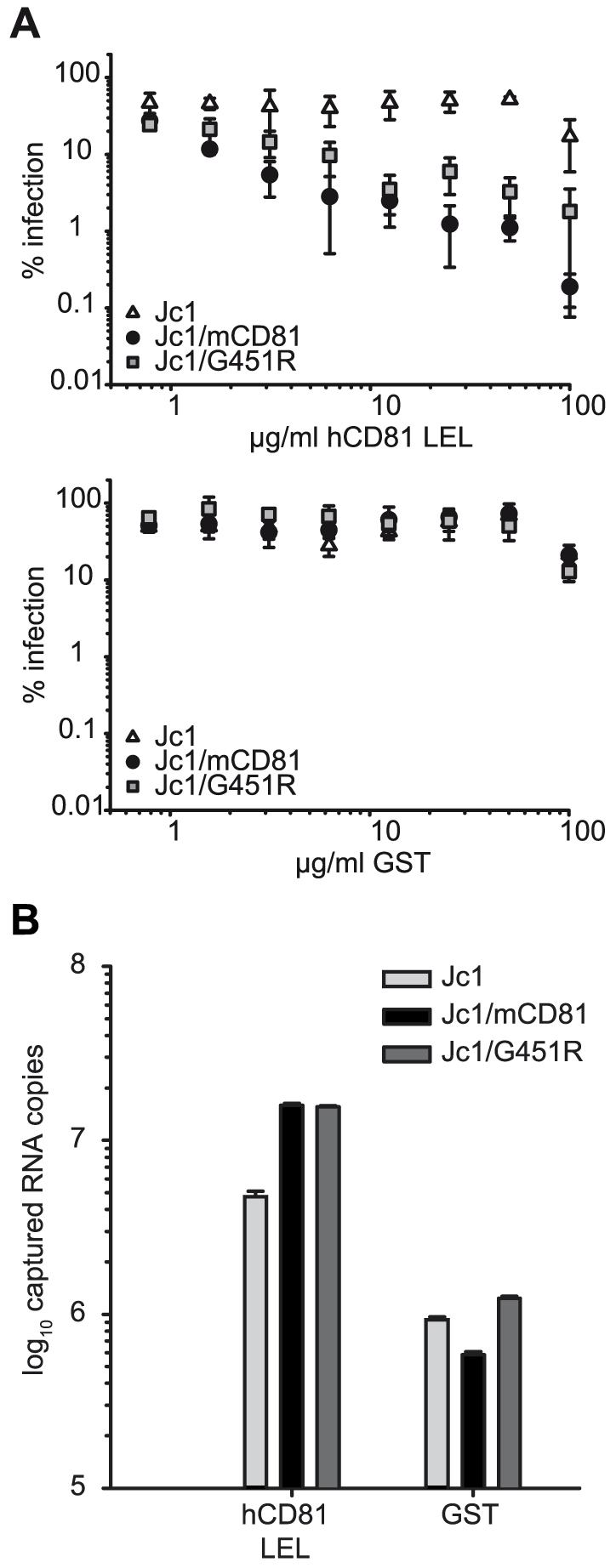
Interaction of HCV wildtype and mutant glycoproteins with recombinant CD81. (A) Neutralization with GST or GST-hCD81 LEL fusion proteins: Cell culture supernatant harvested from Huh-7.5 cells transfected with indicated luciferase reporter virus constructs were preincubated with varying amounts of the indicated recombinant proteins for 30 minutes at room temperature. Subsequently, mixtures were used to inoculate Lunet N hCD81 cells. Cells were lysed 72 h post infection; the firefly luciferase activity was measured and is given in percent relative to control infection in the absence of GST proteins. Mean values of triplicate measurements including the standard deviation are given. (B) Viruses from cell culture supernatant of Huh-7.5 cells transfected with the indicated RNA genomes and normalized for equal amount of core protein were precipitated by GST or human CD81-LEL GST fusion proteins coupled to glutathione agarose beads. The amount of precipitated virus was determined by quantitative RT-PCR.

It is conceivable that improved usage of mouse CD81 by HCV may be linked with augmentation of the affinity between the adapted viral glycoproteins and CD81. Therefore, we assessed whether these mutations altered the interaction between the viral E1/E2 glycoprotein complexes and CD81. To this end, we utilized a recombinant fusion protein between GST and the human CD81 large extracellular loop (LEL) which encompasses the viral binding site and has previously been used to probe this interaction [28], [29]. To distinguish possible changes of virus receptor usage of mouse CD81 adapted glycoproteins from altered receptor usage caused by cell culture adaptation of HCV after long term passage, we created an additional virus mutant in the context of the Jc1 chimera which harbours a single G451R mutation within the E2 protein. This mutation was originally identified in the JFH1 isolate upon long term passage [39] and the G451 position is conserved between the JFH1 isolate and the J6CF strain of HCV as present in the Jc1 chimera. More recently, the G451R mutation was shown to increase the interaction of JFH1 E2 with CD81 and to reduce virus dependence on SR-BI [40]. Unlike Jc1/mCD81, Jc1/G451R still displays a strong preference for infection via human rather than mouse CD81 (Figure S3). Interestingly, when we performed infections in the presence of increasing doses of the GST hCD81 LEL, the fusion protein only poorly neutralized infection by parental Jc1 (Figure 3A). These data indicate that soluble human CD81 does not efficiently compete with cell surface resident CD81 during infection of parental Jc1. In contrast preincubation of Jc1 variants with 10 µg/ml hCD81 LEL reduced infection by these viruses more than 10-fold, suggesting that both adapted variants are more susceptible to inhibition by soluble human CD81 protein (Figure 3A). Similarly, both viruses with adapted glycoproteins were also precipitated more efficiently by hCD81LEL as is evident from 2-3-fold higher amounts of HCV RNA associated with hCD81LEL coated beads incubated with Jc1/mCD81 or Jc1/G451R compared to beads incubated with an equal amount of wildtype Jc1 particles (Figure 3B). Thus, both the cell culture adaptive mutation G451R and the triple mutation identified by us increased interaction with soluble human CD81 possibly by enhancing the exposure of the viral CD81 binding site.

### Mouse CD81 adapted HCV uses human CD81 with increased efficiency

**Figure 4:**
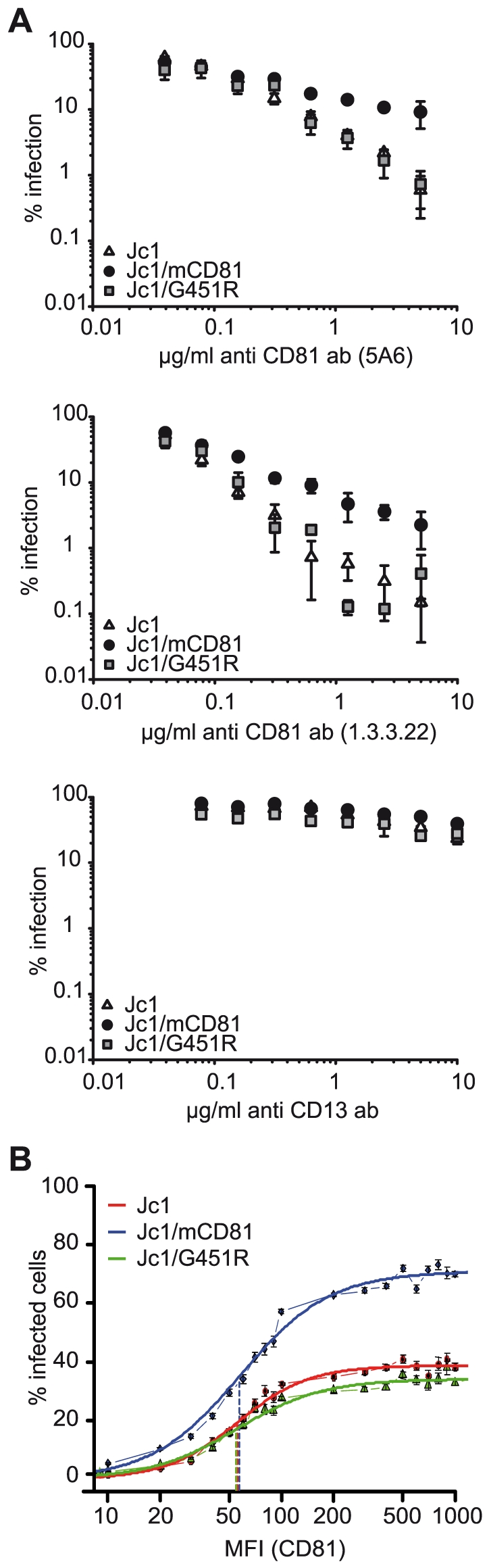
Efficiency of human CD81 usage by Jc1 and Jc1 variants. (A) Neutralization with antibodies specific for human CD81: Indicated viruses (luciferase reporter viruses) were mixed with different concentrations of the human CD81-specific monoclonal antibodies 5A6 or 1.3.3.22. Alternatively, viruses were mixed with CD13-specific control antibodies. Subsequently, mixtures were used to inoculate Lunet N hCD81 cells. Infectivity was determined and expressed as described in the legend of figure 3. (B) Determination of the threshold level of CD81 needed for infection: Naïve Huh7-Lunet cells were mixed with Lunet N hCD81 cells in a ratio of 1∶1 and were infected with indicated Venus Jc1 reporter viruses normalized for equal amounts of core protein. 72 h after infection the cells were harvested, fixed and stained for CD81 with the CD81 specific antibody 5A6 and a secondary antibody coupled to APC. The Venus GFP signal was plotted against the CD81 expression and the percentage of HCV infected cells at a given CD81 cell surface expression was calculated. The graph displays the percentage of HCV infected cells as a function of CD81 expression level plotted as mean fluorescence intensity (MFI). A representative experiment carried out in duplicates is given. Dashed lines indicate the turning points of the individual curves. FACS dot plots of the experiment are shown as supplementary figureS4.

To assess if increased binding of Jc1/mCD81 to human CD81 correlates with altered receptor usage during virus entry, we used two complementary approaches: First, we analyzed susceptibility of Jc1/mCD81 infection to neutralization with anti-human CD81 antibodies (Figure 4A). Second, we quantified the threshold level of human CD81 needed for productive infection of Huh7-Lunet cells (Figure 4B and S4). Interestingly, selectively Jc1/mCD81 was more resistant to neutralization by anti-human CD81 antibodies compared to the parental Jc1 virus and compared to Jc1/G451R (Figure 4A). These results imply that Jc1/mCD81 competes more efficiently with CD81-antibodies for available CD81 binding sites on the cell surface. To determine the minimal CD81 threshold surface expression needed for infection by these viruses, parental Huh7-Lunet cells displaying low to moderate CD81 surface expression were mixed with Lunet N hCD81 cells expressing high levels of CD81 to create a mixed cell population containing cells with highly divergent CD81 abundance at the cell surface. Subsequently these cells were challenged with Venus-GFP-expressing HCV reporter viruses normalized for equal quantity of core protein [41]. Using this approach the relation between CD81 cell surface level and infection efficiency can be quantified using a dual colour FACS analysis [41]. Jc1/mCD81 infected up to 71% of cells with high CD81 expression (ca. 1,000 MFI) compared to Jc1 and Jc1/G451R infecting only 38.9% and 34% of cells with this CD81 abundance (Figure 4B and S4). However, this increased efficiency of Jc1/mCD81 was not accompanied by a reduction of the minimal threshold density of CD81 required for entry since the turning point of the curves which describe the relation between CD81 receptor density and % infected cells was not significantly different between the three viruses compared (Jc1 MFI 55.70; Jc1/mCD81 MFI 57.74; Jc1/G451R MFI 54.90; p-value >0.5). Therefore, we conclude that all three viruses share a similar minimal human CD81 receptor density to permit infection. However, selectively Jc1/mCD81 has attained a higher efficiency of infection in cells displaying CD81 levels in excess of the minimal CD81 level. Together these results argue that Jc1/mCD81 uses human CD81 with elevated efficiency, suggesting either that the avidity (the combined strength of multiple interactions) of the virus for human CD81 has increased and/or that the threshold avidity needed for proper CD81 usage was lowered.

### Adaptation to mouse CD81 modulates usage of human SR-BI and OCLN

**Figure 5:**
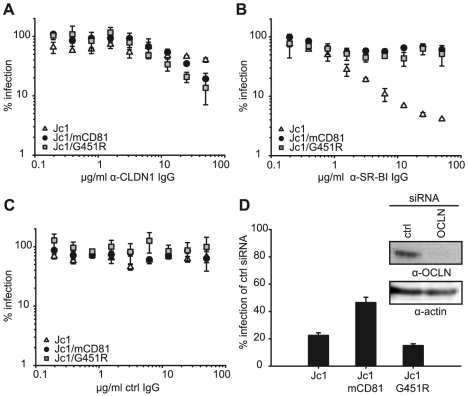
Dependence of wildtype and mutant HCV on SR-BI, CLDN1 and OCLN. (A–C) Neutralisation of indicated viruses with purified immunoglobulins from rats immunized with CLDN1 (A) or SR-BI (B) cDNAs. Immunoglobulins purified from naïve rats served as control (C). Infectivity was determined as described in Figure 3 and is expressed relative to infections conducted in the absence of antibodies. Mean values of triplicate measurements including the standard deviation are given. (D) OCLN expression in Lunet N hCD81 cells was downregulated by transfection with siRNAs specific to OCLN mRNA. A scrambled siRNA was transfected in parallel and served as control. Knock down efficiency was monitored by lysing a fraction of the cells at the time point of inoculation with indicated luciferase reporter viruses. Infectivity was determined 48 h later and is expressed relative to the one measured in cells transfected with the control siRNA. Mean values of triplicates including the standard deviation are shown.

Next, we analyzed if the adaptation of HCV to mouse CD81 has influenced the utilization of human SR-BI, CLDN1 or OCLN. To this end we employed SR-BI- and CLDN1-specific and control antibodies for receptor competition assays comparing parental Jc1 with Jc1/mCD81 and Jc1/G451R (Figure 5A–5C). Since OCLN-specific neutralizing antibodies are not available, we assessed the dependence of these viruses on OCLN surface expression using RNA interference (Figure 5D). Our data indicate that all viruses compared by us need CLDN1 for cell entry, since receptor-specific antibodies inhibited infection by each virus in a dose-dependent fashion (Figure 5A). Dependence of all three viruses on CLDN1 was also confirmed by inoculation of HuH6 cells which express only minimal amounts of CLDN1, rendering them refractory to Jc1 infection [26] and also to both adapted viruses (data not shown). Notably, virus neutralization through SR-BI-specific antibodies was clearly less efficient for Jc1/G451R and Jc1/mCD81 compared to parental Jc1 particles reducing infectivity to approximately 80% only compared to 5%, respectively (Figure 5B). These data confirm the findings of Grove et al., who observed a similar phenotype for JFH1/G451R [40] and indicate that both virus mutants are less dependent on SR-BI for cell entry. Finally, knock down of OCLN reduced efficiency of cell entry for all three viruses, although the reduction was less pronounced for Jc1/mCD81 (p<0.001) (Figure 5D). In conclusion these results suggest that adaptation to mouse CD81 has not only lowered dependence on human CD81 but also on SR-BI and OCLN expression.

### HCV usage of mouse CD81 is accompanied by exposure of neutralizing epitopes and altered requirements for low pH-induced cell entry

Since structural information of the HCV E1/E2 complex is not available, we performed a series of virus neutralization experiments using well defined E2-specific monoclonal antibodies with a conserved linear epitope or conformational binding sites in order to document structural changes of the E1/E2 complex that may be responsible for the phenotypic changes of adapted HCV. More specifically, we used AP33 a mouse monoclonal antibody recognizing a linear epitope within E2 [42], and three human monoclonal antibodies interacting with three distinct conformational domains on the E1/E2 complex designated A, B and C [43], [44]. Antibodies interacting with domain A like CBH4D are non-neutralizing, whereas antibodies to domains B or C (e.g. CBH5 or CBH23, respectively) inhibit E2 binding to CD81 and neutralize infection of HCVpp and HCVcc from genotypes 1 and 2 [45], [46].

**Figure 6:**
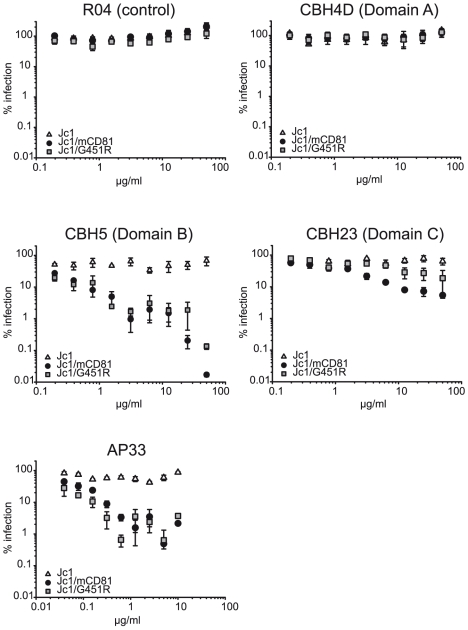
Neutralization of wildtype and mutant HCV with E2-specific antibodies. Given viruses were precincubated for 30 minutes at room temperature with indicated doses of human monoclonal antibodies or the mouse monoclonal antibody AP33. Subsequently, mixtures were used to inoculate Lunet N hCD81 cells. Infectivity was determined as described in figure 3 and is expressed relative to infections conducted in the absence of antibodies. Mean values of triplicates including the standard deviations are given. The RO4 human monoclonal antibody recognizes a cytomegalovirus protein and was used as control.

As expected, even high doses of the non-neutralizing antibody CBH4D and the R04 antibody which recognizes a protein of the cytomegalovirus, did not interfere with infection of all three viruses tested (Figure 6). AP33, CBH5 and CBH23 did not markedly neutralize Jc1. In contrast, these antibodies neutralized Jc1/mCD81 and Jc1/G451R infection in a dose-dependent fashion, indicating that both adapted viruses are much more prone to neutralization by E2-specific antibodies (Figure 6). Notably, domain C antibody (CBH23) neutralized Jc1/mCD81 and Jc1/G451R to a different degree with Jc1/mCD81 being more susceptible to the antibody (IC90 of Jc1/mCD81 and Jc1/G451R: 12.9 µg/ml and 157.3 µg/ml, respectively, p<0.001). In contrast, both viruses were similarly neutralized with the AP33 antibody (IC90 of Jc1/mCD81 and Jc1/G451R: 0.27 and 0.07 µg/ml, respectively, p = 0.28) and with the domain B antibody (CBH5; IC90 of Jc1/mCD81 and Jc1/G451R: 0.18 and 0.07 µg/ml, respectively, p = 0.18). The differential susceptibility to neutralizing antibodies was not due to overtly different buoyant density of these viruses (Figure S5) and is indicative of distinct structures of the respective viral glycoprotein complexes. These in turn are likely responsible for the different phenotypes regarding host entry factor usage and tropism.

**Figure 7:**
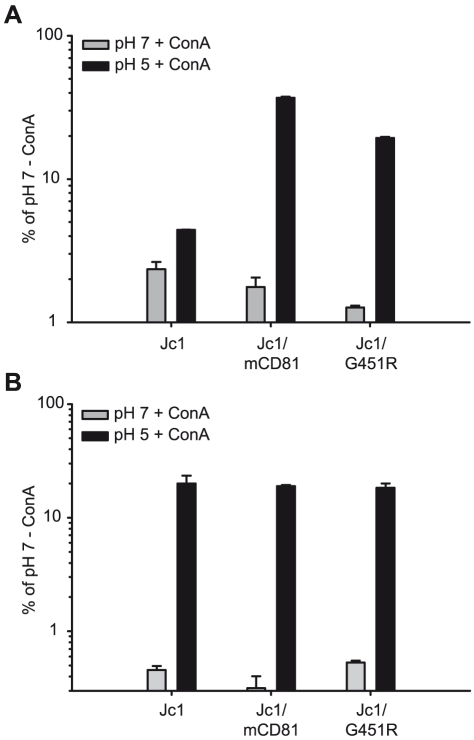
Induction of HCV cell entry by low pH treatment. Lunet N hCD81 cells were pretreated with Concanamycin A (ConA) at a concentration of 5 nM to prevent acidification of cellular endosomes and thus the natural route of HCV cell entry. Pretreated cells were inoculated with given F-Luc Jc1 variants in the presence of ConA at 4°C for 2 h. Cells were washed twice with cold PBS either directly followed by an incubation in citric acid buffer of pH 7 or pH 5 for 5 min at 37°C (A). Alternatively, the cells were first shifted to a temperature of 37°C and were incubated for 1 h in the continued presence of ConA before they were incubated with citric acid buffer of pH 5 or pH 7 for 5 minutes (B). Thereafter, cells were cultured an additional 3 h in the presence of 5 nM ConA before the medium was changed. Cells were lysed after 48 h; the luciferase signal was measured and is expressed relative to control infections conducted in the absence of ConA and where the cells were incubated with citric buffer of pH 7. Mean values of triplicate measurements including the standard deviation are given.

After cell surface binding and virus internalization into clathrin-coated pits, HCV infection is mediated by conformational changes of the viral glycoprotein complex E1/E2 which are triggered by low pH [24]. Notably, the responsiveness of HCV to low pH-triggered fusion seems to change over time: First, unlike for alphaviruses which also enter host cells in a low pH-dependent fashion and which are inactivated by low pH-treatment in solution, HCV resists such treatment and retains infectivity [24]. Second, exposing HCV particles directly after binding to permissive cells at 4°C, a temperature which allows binding of the viral particles, but no further steps of the entry process, to a low pH wash does not efficiently initiate infection. In contrast, chasing these particles for 1 h at 37°C increases their sensitivity to low pH which may indicate that HCV requires certain additional stimuli like for instance receptor interactions to prepare the virus for low pH-triggered fusion and infection [24]. Given these findings, we hypothesized that adaptation to mouse CD81 may have selected for altered E1/E2 complexes that are more responsive to conformational changes triggered by receptor interactions and/or low pH, thus permitting entry through use of an entry factor of non-human origin with poor binding affinity for HCV. To test this hypothesis we compared the responsiveness of Jc1, Jc1/mCD81 and Jc1/G451R to low pH-treatment directly after cell surface binding and 1 h later following the protocol described by Tscherne et al. [24]. Importantly, infection via acidified endosomes is blocked throughout these experiments by treatment of target cells with concanamycin A (ConA). Since this drug inhibits endosomal V-ATPases, acidification of endosomes is inhibited thus ablating infection through the natural endosomal route [24]. In agreement with the observation by Tscherne et al., treatment with pH 5 directly after virus binding at 4°C stimulated Jc1 infection with very low efficiency, reaching only ca. 4% of the infection attained when acidification of endosomes was not blocked by addition of ConA and viruses were treated with pH 7 only (Figure 7A). Infectivity of both Jc1/mCD81 and Jc1/G451R was comparable to Jc1 when washed with pH 7 ranging between 1 and 3% of control infections in the absence of ConA. However, both viruses strongly responded to the pH 5 wash already directly after virus binding reaching up to 30% and 20% of the untreated control. After incubation of cell-bound HCV for 1 h at 37°C also Jc1 particles could be stimulated to infect ConA-treated cells (Figure 7B). Therefore, unlike wildtype HCV both adapted viruses can be triggered to infect ConA-treated cells already directly after cell surface binding suggesting that the requirements for induction of infection by low pH have been modified.

### Adaptive mutations within E1/E2 permit HCV infection of mouse cells

**Figure 8:**
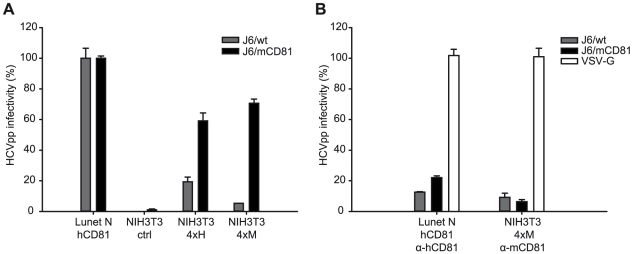
CD81 dependent infection of NIH3T3 cells expressing human or mouse entry factors by HCVpp. (A) NIH3T3 cell derivatives transduced with empty expression vectors (NIH3T3 ctrl) or expressing either all four human or mouse HCV entry factors (NIH3T3 4xH, NIH3T3 4xM, respectively) were challenged with concentrated retroviral pseudoparticles coated with Vesicular stomatitis virus G-protein (VSV-G), wildtype J6CF glycoproteins (J6/wt) or with mouse CD81 adapted J6CF glycoproteins (J6/mCD81). Lunet N hCD81 cells were inoculated in parallel. Productive infection was determined by FACS analysis based on Venus-GFP expression derived from the retroviral vector. The HCVpp signal was normalized to VSV-Gpp infectivity and is expressed relative to HCVpp infectivity determined on our human reference cell line Lunet N hCD81. Mean values including standard deviations of a representative experiment of five independent experiments are given. (B) Pseudoparticle infection was neutralized with human and mouse CD81 specific antibodies (5A6 and EAT1 respectively, both Santa Cruz) at the time of inoculation. Note that infections presented in (A) and (B) were performed in parallel. Control infections performed in the absence of CD81-specific antibodies are displayed in panel (A). Dot Plots of the experiment are displayed in supplementary figure S7.

Finally, we analyzed if the mutations selected by adaptation of Jc1 to mouse CD81 permit HCV infection of mouse cells in the absence of human factors. For these experiments we chose NIH3T3 cells which become susceptible to HCVpp by ectopic expression of human SR-BI, CD81, CLDN1 and OCLN [8]. While these cells express endogenous mouse CD81 and mouse SR-BI, we were unable to detect mouse CLDN1 and OCLN (Figure S6). Given these circumstances, we created NIH3T3-derived cell lines which ectopically express all four human or mouse HCV entry factors, designated NIH3T3-4xH or NIH3T3-4xM. Since mouse cells support very low HCV RNA replication, we used murine leukemia virus-based HCV pseudoparticles (HCVpp) transducing a Venus-GFP reporter gene to quantify HCV cell entry into these cells. As expected, HCVpp with wildtype or mouse CD81 adapted glycoproteins were unable to infect control NIH3T3 cells but efficiently entered Lunet N hCD81 cells (Figures 8A and S7A). Ectopic expression of human HCV entry factors in NIH3T3 cells rendered these cells permissive to both wildtype HCV and mouse CD81 adapted HCVpp, reaching infectivities as high as ca. 20% and 60% of the infectivity in Lunet N hCD81 cells, respectively. In contrast, NIH3T3 cells expressing mouse entry factors (NIH3T3 4xM) were only efficiently infected with HCVpp carrying mouse CD81 adapted glycoproteins. Importantly, HCVpp coated with mouse CD81 adapted glycoproteins infected NIH3T3 cells with human or mouse entry factors with comparable efficiency and infectivity of HCVpp was efficiently neutralized by anti-human or anti-mouse CD81 antibodies in the respective cell lines (Figures 8B and S7B). In agreement with our data described above, HCVpp coated with wildtype J6 glycoproteins were more prone to neutralization by anti-CD81 antibodies compared to J6/mCD81-coated HCVpp. In conclusion these results indicate that three adaptive mutations within HCV E1/E2 proteins are sufficient to permit efficient HCV entry into mouse cells in the absence of human factors. We were unable to detect productive entry of HCVpp with wildtype or mouse CD81-adapted glycoproteins into primary mouse hepatocytes. We cannot exclude that dominant restriction factors expressed in these cells may limit cell entry even of mouse adapted HCVpp. However, we observed very low expression of mouse CLDN1 and undetectable quantities of mouse OCLN in these cells (Fig. S8) which very likely precludes efficient HCV cell entry.

## Discussion

In this study we took advantage of a human liver cell line expressing mouse in place of human CD81 to select for a population of HCV that utilizes this entry factor with high efficiency for infection of host cells. Employing reverse genetics we showed that the ability to utilize mouse CD81 was conferred by a combination of three mutations within the viral surface proteins E1 and E2.

In principle, change of receptor usage mediated by these mutations may reflect an adaptation of the viral CD81 binding site to accommodate the mouse entry factor and to enhance binding affinity. However, neither these residues nor the domains in which they are located in have previously been implicated in the virus-CD81 interaction. Two of the identified mutations (V388G and M405T) are part of the so called hypervariable region 1 (HVR1). This highly variable domain at the N-terminus of E2 has been implicated in the interaction with SR-BI [47], [48] and is dispensable for binding of soluble E2 to CD81 [31]. In fact, deletion of HVR1 enhances binding of soluble E2 to soluble or cell surface-bound CD81 [31]. In addition viruses lacking HVR1 are only moderately impaired in entry and remain susceptible to neutralization by CD81-specific antibodies [48], thus strongly suggesting that the HVR1 is dispensable for the interaction with CD81. Consequently, it is unlikely that the HVR1 is part of the viral CD81 binding site and that the adaptive mutation within this domain directly enhance the binding to mouse CD81.

Alternatively, adaptive changes may act indirectly by eliminating obstructions that otherwise sterically prevent access of mouse CD81 to the viral CD81 binding site. In the absence of structural information of the viral E1/E2 complex we probed the influence of these mutations on the interaction with CD81 using soluble human CD81 and CD81-specific antibodies. To phenotypically distinguish adaptation to mouse CD81 from cell culture adaptation we compared the mouse CD81-adapted virus to a recently described cell culture-adaptive mutation within E2 (G451R) that is known to modulate SR-BI and CD81 receptor usage and virus neutralization [49]. Interestingly, both mutant viruses interacted more efficiently with soluble human CD81-LEL and they were both more susceptible to neutralization by this protein (Figure 3). Previously, Flint et al. were unable to detect binding of HCV E2 protein to soluble mouse CD81 [25]. Our attempts to detect changes of HCV binding to soluble mouse CD81 did not yield conclusive results precluding a definite answer if the affinity to mouse CD81 was changed by the adaptation (data not shown). Therefore, it remains to be determined if parental or adapted HCV binds mouse CD81. Nevertheless, these results indicate that both virus mutants have a more accessible binding site for human CD81 which likely facilitates interaction and neutralization. Such a more open conformation may also assist interaction with mouse CD81. Notably, also the mouse CD81 adapted virus remains dependent on CD81 since it does not infect cells devoid of CD81. Therefore, either the adapted HCV indeed does interact with mouse CD81 or it utilizes an essential function of mouse CD81 as entry factor even in the absence of direct binding. As CD81 is a member of the protein family of tetraspanins, which assemble multi-protein complexes within membranes, such a function could for instance be the coordination of additional HCV entry factors into a functional receptor complex. In line with this notion, a direct interaction between CD81 and CLDN1 has been reported [50], [51].

Interestingly, the mouse CD81 adapted virus was more resistant to neutralization by CD81-specific antibodies and infected cells carrying a given abundance of human CD81 at their surface with increased efficiency compared to wildtype and the Jc1/G451R variant HCV (Figure 4). These findings suggest that only the mouse CD81-adaptive changes have enhanced human CD81 receptor usage for infection. Notably, this change however was not accompanied by a reduction of the minimal threshold CD81 density needed for viral entry (Figure 4B). It is worth mentioning here that Grove et al. noted decreased neutralization of JFH1 carrying the G451R mutation by CD81-specific antibodies [49]. Although we do not know the reason for this discrepancy it is possible that the phenotype of this mutation at least with regard to CD81 usage may differ dependent on the strain of glycoproteins (J6 and JFH1, respectively).

Receptor competition experiments with antibodies against SR-BI and CLDN1 revealed a similar behaviour of the mutant viruses, both exhibiting a decreased SR-BI dependence, while CLDN1-dependence was indistinguishable between both mutant viruses and wildtype HCV (Figure 5). Finally, when reducing the number of available OCLN molecules by RNA interference, both wild type and the G451R adapted virus were clearly more inhibited than Jc1/mCD81. These data indicate that both cell culture adaptation as well as adaptation to mouse CD81 caused reduced SR-BI dependence, however only the latter procedure at the same time also reduced dependence on human CD81 and OCLN.

A third possible mechanism that may permit utilization of mouse CD81 for entry is through facilitating essential conformational changes of the glycoprotein complex. This could for instance be mediated by an opening of the overall structure of the viral surface proteins. Our neutralization experiments with E2-specific antibodies revealed a much enhanced susceptibility to neutralization for both mutant viruses. Interestingly, susceptibility to domain C-specific antibodies was more pronounced for the mouse CD81 adapted virus than for Jc1 with the G451R mutation, indicating distinct conformations of the virus particle-resident E2 proteins at this domain. These findings are both suggestive of gross conformational changes and of increased exposure of key neutralizing epitopes. Since these changes were not accompanied by an altered distribution of these viruses in density gradients, we believe that they are not due to differential interaction with lipoproteins. As additional indicator for altered surface protein conformation and as a measure for viral responsiveness to triggers of cell membrane fusion, we compared the ability of wildtype and mutant HCV to infect host cells stimulated by a low pH wash. In agreement with previous findings, wildtype HCV was refractory to a low pH wash immediately after binding to the cell surface at 4°C and only infected cells after an incubation of 1 h at 37°C [24]. In contrast, both mutant viruses could be induced to infect cells by low pH directly after binding. This suggests that the requirements for induction of pH-dependent fusion have changed for both viruses.

Since the structure of the HCV E1/E2 complex is unknown it is difficult to finally conclude which of the above mentioned mechanisms that are certainly not mutually exclusive, is responsible for the efficient utilization of mouse CD81. Our data support a model where this combination of mutations leads to an opening of the glycoprotein complex. This “unlocked” structure increases exposure of the CD81 binding site and likely in turn interaction with soluble CD81. Moreover, it also facilitates conformational changes which may permit the virus to utilize a weak CD81 binding (e.g. due to interaction with CD81 from non-human species which bind with low affinity) for its entry process. In addition, such a conformational change may contribute to decreased dependence on SR-BI and OCLN and a lower threshold for pH-triggered cell entry. This interpretation may also explain why these mutations selected on mouse CD81 increase the usage of rat and hamster CD81 to a comparable degree (approximately 100-fold). Given that these forms of CD81 differ from each other by a number of amino acids within the viral binding site [25], this would have been unlikely, if the viral binding site had specifically adjusted to mouse CD81.

Finally, we show that HCV J6-derived glycoproteins adapted to mouse CD81 permit infection of mouse cells in the absence of human factors. Thus, three adaptive mutations within E1 and E2 are sufficient to overcome the barrier for cell entry into mouse cells. In our view, this finding lends further support to the notion that these mutations may facilitate receptor-dependent conformational changes of the glycoproteins thus permitting the use of mouse CD81 and ultimately infection of cells via solely mouse factors. Previous reports have established that besides CD81 at least OCLN poses a strong species barrier to HCV infection [8], [25]. In addition, evidence was reported that sub-optimal usage of mouse CLDN1 and SR-BI may also limit efficient infection of mouse cells [26], [27]. Therefore, poor entry of HCV into mouse cells seems to be determined by multiple entry factors, making it difficult to envision that mere adaptation of the viral CD81 binding site would be sufficient to allow usage of mouse SR-BI, OCLN, and CLDN1. Notably, while the G451R mutation only reduced SR-BI-dependence, the mouse CD81 adaptive mutations at the same time also reduced CD81- and OCLN-dependence which may be an important prerequisite for the virus to utilize the foreign entry factors which possibly interact with lower affinity.

Likely due to very low RNA replication of HCV in mouse cells we were unable to observe productive infection of mouse cells by Jc1/mCD81 (data not shown). In turn these results highlight that additional host factors limit propagation of HCV in mouse cells downstream of virus cell entry. Nevertheless, our findings are proof of concept that a limited number of mutations can be sufficient to adapt the virus for usage of foreign host factors or possibly even host factor complexes and therefore encourage future efforts to adapt HCV for growth in non-human cells. Combined with genetic approaches and xenotransplantation [52] such efforts may ultimately lead to the development of immune competent small animal models for research of HCV pathogenesis, immune control as well as vaccine and drug development.

## Materials and Methods

### Plasmids

The plasmids pFK Jc1, pFK-Luc-Jc1 and pFK-Venus-Jc1 have been described earlier [37], [41], [48]. The constructs pFK Jc1/L216F, pFK Jc1/V388G, pFK Jc1/M405T, pFK Jc1/N767D, pFK Jc1/V388G/M405T, pFK Jc1/L216F/V388G/M405T (equivalent to pFK Jc1/mCD81) and pFK Jc1/L216F/V388G/M405T/N767D were created via PCR-based site-directed mutagenesis. PCR inserts were sequenced to exclude off site mutations. The constructs pcDNA3ΔcE1E2J6/mCD81 (containing L216F, V388G and M405T) and pcDNA3ΔcE1E2J6/G451R are derivatives of pcDNA3ΔcE1E2J6 that has been described recently [26]. pRV Venus was created by insertion of the gene encoding Venus-GFP [53] into the retroviral vector pczCFG5IZ [54]. Lentiviral plasmids pWPI humanCD81 BLR, pWPI ratCD81 BLR, pWPI hamsterCD81 BLR, pWPI mouseCD81 BLR, pWPI humanCD81 Gun, pWPI mouseCD81 Gun, pWPI humanOCLN Gun, pWPI mouseOCLN Gun, pWPI humanSR-BI Gun, pWPI mouseSR-BI Gun, pWPI human CLDN1 BLR and pWPI mouse CLDN1 BLR encode the human, rat, hamster or mouse orthologs of the four HCV receptors in the context of the self inactivating lentiviral vector pWPI [55]. In this context the transgene is expressed by an internal human elongation factor 1 alpha (EF1-α) promoter. The transcribed unit also contains an IRES element from EMCV that allows internal initiation of translation of a blasticidin S deaminase of *Aspergillus terreus* or a GFP-ubiquitin neomycinphosphotransferase fusion protein (Gun) as selectable markers. Exact cloning strategies and primer sequences can be obtained on request.

### Cell culture

Huh-7.5, Huh7-Lunet, HuH6, 293T and NIH3T3 cells were cultured in Dulbecco's modified Eagle medium (DMEM; Invitrogen, Karlsruhe, Germany) supplemented with 2 mM L-glutamine, non-essential amino acids, 100 U of penicillin per ml, 100 µg of streptomycin per ml, and 10% fetal calf serum (DMEM complete) at 37°C and 5% CO_2_. Lunet N cells were generated by FACS sorting of CD81 low expressing cells within the Lunet cell population and subsequent subcloning by limiting dilution. Three clones were analyzed further with regard to CD81 expression and permissiveness for HCV RNA replication (clones #3, #4, and #7). Of these subclones, number #4 and #7 were described recently [36] and subclone #3 was used throughout this study and was designated as Lunet N.

Stable cell lines were generated via lentiviral gene transfer as described recently [56] using the three plasmids pCMVΔR.74 [57], a pWPI derivative (either coding for a resistence against blasticidine (blasticidine S deaminase; BLR) of *Aspergillus terreus* or a GFP-ubiquitin-neomycin fusion protein (Gun) and the respective gene of interest) and pcz VSV-G [58] in a ratio of 3∶3∶1. Selection was carried out in the presence of either 5 µg/ml Blasticidin or 0,75 mg/ml G418.

### Viruses and HCV pseudoparticles

HCVcc particles and firefly luciferase HCV reporter viruses were generated as reported previously [48]. In brief, plasmid DNA was linearized and transcribed into RNA, which was then electroporated into Huh-7.5 cells. Virus-containing culture fluids of transfected cells were harvested 48 h and 72 h after transfection.

Luciferase reporter virus infection assays were carried out and analyzed as described [48] using Lunet N hCD81 cells as target cells. Wildtype HCV particles were titrated by using a limiting dilution assay [59]. The 50% tissue culture infectious dose (TCID_50_) was calculated based on the methods described by Spearman and Kärber [60], [61].

Murine leukemia virus (MLV)-based pseudotypes carrying vesicular stomatitis virus glycoproteins (VSV-G) or the HCV J6-derived E1 and E2 proteins as well as their mouse CD81 adapted derivatives (J6/mCD81) were generated by cotransfection of 293T cells. Briefly, 1.2×10^6^ 293T cells were seeded into 6-cm-diameter plates 1 day before transfection with 2.6 µg envelope protein expression construct pczVSV-G, pcDNA3ΔcE1E2-J6, pcDNA3ΔcE1E2-J6/mCD81 or an empty-vector control, 2.6 µg MLV Gag-Pol expression construct pHIT60, and 2.6 µg firefly luciferase transducing vector by using Lipofectamine 2000 (Invitrogen). The medium was replaced 6 h after transfection, and the supernatants containing the pseudoparticles were harvested 48 h later. The supernatants were cleared of cells by passage through a 0.45-µm-pore-size filter, concentrated ca. 10-fold by ultrafiltration with Amicon Ultra centrifugal filter units (molecular weight cut off 100 kDa, Millipore, Schwalbach, Germany) and used to infect Lunet N hCD81 and NIH3T3 cells and its derivatives expressing HCV entry factors of human and mouse origin. For infection, target cells were seeded at a cell density of 2×10^4^ per well of a 48-well plate 24 h prior to inoculation. Cells were inoculated for 4 h with retroviral pseudoparticles and cells were cultured an additional 48 h prior to harvesting and fixation in 1% paraformaldehyde (PFA) w/v. Venus GFP-expression as marker for productively infected cells was quantified using a FACS-Calibur flow cytometer (Becton Dickinson, Heidelberg, Germany) and the Flow Jo software package. Raw FACS scatter plots of one representative experiment of five independent repetitions are depicted in supplementary Figure S7. A representative experiment carried out in duplicates from 5 independent experiments is depicted in Figure 7. The background Venus-GFP signal from non-enveloped pseudoparticles (Env-pp) was first subtracted from the VSV-Gpp and HCVpp signals. The HCVpp signal was then normalized to VSV-Gpp infectivity [(HCVpp-NE)/(VSVG-NE)]. Finally, these values were expressed relative to HCVpp infectivity determined on our human reference cell line Lunet N hCD81. As a result, the data reflect the relative ability of HCVpp to infect mouse NIH3T3 cells expressing different human or mouse entry factors compared to highly permissive human host cells (Lunet N hCD81).

### CD81 interaction assays

CD81 LEL GST fusion proteins were prepared as described [25]. In brief the expression constructs were transformed in *E.coli rosetta gami*. A 1 l culture of the bacteria was grown to an OD_592_ of 0.8 at 37°C and induced to express the GST fusion proteins with 1 mM IPTG (Sigma-Aldrich, Steinheim, Germany) at RT over night. Bacteria were first lysed with 1 mg/ml chicken egg lysozyme (Sigma) and 500 U benzonase in PBS and in a second step with 0.5% nonidet P-40 (NP40) in PBS (lysis buffer) the supernatant was incubated with 2 ml of a 1∶1 slurry glutathione agarose (Sigma) for 2 h at RTunder gentle agitation washed three times with 25 ml lysis buffer and afterwards the bound proteins were eluted with 100 mM Glutathione (Sigma-Aldrich, Steinheim, Germany) in PBS and dialysed in PBS over night. Concentrations were determined by Bradford.

To determine the interaction between the recombinant CD81 protein and virus particles, 500 µl virus-containing culture fluid harvested 48 h post transfection of Huh-7.5 cells and normalized for the amount of Core protein were preincubated with 50 µl glutathione agarose beads (1∶1 slurry). These preadsorbed virus preparations were incubated with 50 µl glutathione agarose beads that had been coupled with 10 µg recombinant GST-CD81-LEL proteins and the mixture was incubated on a rotator at 4°C over night. Subsequently, beads were washed 3 times with PBS and then resuspended in 200 µl of PBS. The amount of bead-associated HCV core protein was determined by a commercial core-specific ELISA (Wako Chemicals, Neuss, Germany). HCV RNA associated with beads suspended in 100 µl PBS was extracted using the Nucleo Spin RNAII kit (Macherey-Nagel, Düren, Germany) as recommended by the manufacturer. Two microliters of the RNA sample were used for quantitative RT-PCR analysis using a LightCycler 480 (Roche, Mannheim, Germany). HCV-specific RT-PCRs were conducted in duplicate employing a one-step RT-PCR LightCycler 480 RNA Master Hydrolysis Probes kit (Roche, Mannheim, Germany) and the following JFH1-specific probe (TIB Molbiol, Berlin, Germany) and primers (MWG-Biotech, Martinsried, Germany): A-195 [5′-6FAM (6-carboxyfluorescein)-AAA GGA CCC AGT CTT CCC GGC AAT T-TAMRA (tetrachloro-6-carboxyfluorescein)-3′], S-146 (5′-TCT GCG GAA CCG GTG AGT A-3′), and A-219 (5′-GGG CAT AGA GTG GGT TTA TCC A-3′).

### Neutralization assays

Luc-Jc1 viruses were incubated with different concentrations of the indicated antibodies for 30 minutes at RT prior to inoculation of Lunet N hCD81 cells which had been seeded at a density of 0.7×10^4^ cell/96-well the day before. Each neutralization assay was conducted in triplicates with a total volume of 100 µl/well. 4 h after inoculation with the virus/antibody mixture another 100 µl of medium was added. Anti-E2 neutralization assays were conducted using the human E2-specific mAbs CBH4D, CBH23, CBH5, and the R04 antibody which is directed against a cytomegalovirus protein as control [46], [62], and the E2-specific mouse monoclonal antibody AP33 [63]. For receptor neutralization assays the mouse monoclonal anti CD81 antibody 5A6 (Santa Cruz, Santa Cruz, CA) and 1.3.3.22 (Ancell, Bayport, MN) or anti-CD13 (Beckton Dickenson, Heidelberg, Germany) were utilized. IgGs from polyclonal rat sera specific to SR-B1 and CLDN1, raised by genetic immunization of Wistar rats with a plasmid expressing the respective full length cDNA, were produced and purified as described [64], and employed at the indicated concentrations starting with 50 µg/ml.

### RNA interference

SiRNA pools from Dharmacon (Lafayette, CO) specific to human OCLN were transfected via reverse transfection according to the manufacturer's instructions using RNAiMax (Invitrogen) into Lunet N hCD81 cells. Briefly, 150 pmol siRNA were mixed with 200 µl OptiMEM in a 12-well, and 1.5 µl of RNAiMax were added. A total of 6×10^4^ Lunet N hCD81 cells suspended in 1 ml of plain medium with 10% FCS were seeded on top. Knock down efficiency was analyzed 48 h after transfection via Western Blot directly at the time of inoculation with HCV luciferase reporter viruses. Infection efficiency was determined 48 h later using luciferase assays as described above.

### Initiation of HCV infection by low pH wash

The assay was conducted as described earlier [24], [65]. Lunet N hCD81 cells were seeded at a density of 2.5×10^5^ cells/6-well. 24 h later, cells were pretreated with Concanamycin A (ConA; 5 nM) for 1 h at 37°C. Subsequently, cells were shifted to 4°C and were then infected with F-Luc Jc1 variants in the presence of ConA for 2 h at 4°C. Cells were washed twice with cold PBS either directly followed by an incubation in citric acid buffer (McIllivaine's buffer system pH 7 or 5) for 5 min at 37°C. Alternatively, after virus binding at 4°C, cells were first shifted to 37°C for 1 h under continued presence of ConA to 37°C, and only then incubated with citric acid buffer as described above. In both protocols, cells were maintained for another 3 h in medium containing 5 nM ConA and then the medium was replaced. Cells were lysed after 48 h and the luciferase signal was measured. Luciferase values are expressed relative to control infections which were conducted in the absence of ConA and with an incubation step with citric acid buffer of pH 7.

### Densitiy gradient

HCV particles were harvested 48 h after electroportation of Huh-7.5 with the indicated viral RNA. 1 ml of virus containing supernatant was mixed with 2 ml of a 60% (w/v) iodixanol stock solution (Optiprep Axis Shield, Oslo, Norway) and layered under a 0–30% iodixanol gradient. Gradients were centrifuged at 154,000×g for 18 h and afterwards 1 ml fractions were collected from bottom to top. The density of the different fractions was determined via refractometry, the infectivity was determined in limiting dilution assay and the amount of particles was assessed by the determination of RNA copies as described above.

### Flow cytometry

5×10^5^ cells were stained with antibodies specific for hCD81 (5A6, 1∶200, Ancell, Bayport, MN) for mCD81 (EAT2-PE, 1∶20, Santa Cruz, Santa Cruz, CA) or with a polyclonal rabbit serum directed against SR-BI (Novus Biologicals, Littleton, CO 1∶50) in PBS containing 2% FCS for 30 minutes on ice and washed with PBS. In case of noncoupled primary antibodies bound antibodies were detected with secondary antibodies coupled to phycoerythrin or allophycocyanin (1∶200, eBioscience, San Diego, CA) for 30 minutes on ice. After a next washing step the cells were analyzed with a FACS Calibur (Becton Dickinson, Heidelberg, Germany) and the results were analyzed using the Flow Jo Software.

### Western blot analysis

Cells were washed with PBS and lysed in RIPA buffer (0.3 M NaCl, 20 mM TrisHCl pH 8, 1% Sodium dexoxycholat; 0.1% SDS and 1% Triton) for 30 minutes on ice. The total protein content was determined by Bradford assay. Equal protein amounts for each sample were mixed with 2× denaturing protein sample buffer (200 mM Tris-HCl [pH 8.8], 5 mM EDTA, 0.1% bromophenol blue, 10% sucrose, 3.3% sodium dodecyl sulfate [SDS], 2% 2-mercaptoethanol [2-ME]) for 5 minutes at 98°C, loaded onto a 12.5% SDS-gel and resolved by electrophoresis. Subsequently, proteins were transferred with a semiydry blotter to a polyvinylidene difluoride membrane. The membrane was blocked with 5% milk in PBS containing 0.5% Tween (PBS-T) for 1 h at RT. CLDN1, OCLN and actin were detected with specific monoclonal antibodies (Zymed, San Francisco, CA and Sigma-Aldrich, Steinheim, Germany) and a secondary antibody coupled to the horseradish peroxidase (Sigma-Aldrich, Steinheim, Germany). The antibody signal was detected with the ECL Plus detection system (GE Healthcare, Freiburg, Germany).

### Statistical analysis

Comparison of knock down of OCLN reduced entry of Jc1, Jc1/G451R, and Jc1/mCD81 were compared with one-way analysis of variance of hill functions. Similarly, the proportion of infected cells according to their MFI is fitted by nonlinear weighted least squares. IC90 values from neutralization experiments were obtained and compared by nonlinear least squares fit of hill functions. All tests were two-sided and p-values below 5% are considered significant.

## Supporting Information

Figure S1 Characterization of Lunet N cells. (A) FACS analysis for the expression of human and mouse CD81 and SR-BI of Lunet N, Lunet N mCD81 and Lunet N hCD81 cells. Human CD81 was detected with the mouse monoclonal antibody 5A6 (Santa Cruz) and a secondary antibody coupled to APC. Mouse CD81 was detected with the monoclonal hamster antibody EAT2 directly coupled to PE (Santa Cruz) and SR-BI was detected with a polyclonal rabbit serum (Novus Biologicals) and secondary antibodies coupled to Alexa488 (Invitrogen). (B) Western Blot analysis of the tight junction proteins CLDN1 and OCLN (both antibodies from Zymed). Actin expression was determined to ensure equal sample loading. (C) HCV RNA replication in transfected Lunet N cells. The different Lunet N cells were transfected with F-Luc Jc1 RNA and lysed at the indicated time points to measure the reporter activity. To normalize for different transfection efficiency values are expressed relative to the luciferase activity determined 4 h after transfection. Mean values of duplicate measurements are given. (D) Infectivity of HCV in different Lunet N cells. Virus supernatants harvested from Huh-7.5 cells 48 h after transfection were titrated using the limiting dilution assay (TCID_50_) on the indicated Lunet N cell lines. The dashed line indicates the detection limit of the limiting dilution assay. Epo, electroporation.
(1.36 MB EPS)

Figure S2 Adaptation of HCV to mouse CD81 through serial passage in Lunet N mCD81 cells. Cell free culture fluids were collected at indicated steps of the cell culture adaptation procedure and frozen. At the end of the experiments, all samples were titrated in parallel using Lunet N, Lunet N mCD81 and Lunet N hCD81 as target cells. The dashed line indicates the detection limit of the limiting dilution assay.
(0.32 MB EPS)

Figure S3 CD81 usage by Jc1/G451R. Huh-7.5 cells were transfected with Jc1/wt, Jc1/mCD81 or Jc1/G451R RNA and the cell culture supernatant was titrated by the limiting dilution assay on Lunet N hCD81 or Lunet mCD81 cells. The dashed line indicates the detection limit of the limiting dilution assay.
(0.29 MB EPS)

Figure S4 Infection of Lunet cells with diverse CD81 expression with Venus-GFP reporter viruses. Naïve Huh7-Lunet cells were mixed with Lunet N hCD81 cells in a ratio of 1∶1 and were seeded one day prior to infection with Venus Jc1 reporter viruses normalized for equal amounts of core protein. 72 h after infection the cells were harvested, fixed and stained for CD81 with the CD81 specific antibody 5A6 and a secondary antibody coupled to APC. The Venus GFP signal was plotted against the CD81 expression. Mixed Lunet cells were infected with Venus Jc1 reporter viruses bearing the adaptive mutations and normalized for the amount of Core protein. Depicted are representative dot plots of uninfected cells or cells infected with the indicated Venus Jc1 reporter viruses.
(1.18 MB EPS)

Figure S5 Biophysical properties of HCV particles. Cell culture supernatants containing virus particles harvested 48 h after transfection of given HCV genomes were layered under a linear iodixanol gradient. 10 fractions were collected after ultracentrifugation and analyzed for the infectivity on Lunet N hCD81 cells. The density of each fraction was determined by refractometry. The infectivty (A) and the HCV RNA copy numbers (B) in each fraction were determined by the limiting dilution assay and quantitative RT-PCR, respectively. The specific infectivity (C) was calculated for each fraction and is expressed relative to a single HCV RNA genome.
(0.42 MB EPS)

Figure S6 Receptor expression on NIH3T3 cells stably expressing HCV entry factors from mouse or human origin. (A) FACS analysis for the expression of human and mouse CD81 and SR-BI on the surface of NIH3T3 control cells transduced with the empty expression vector (NIH3T3 ctrl), NIH3T3 cells transduced with the four human entry factors(NIH3T3 4xH) or the four mouse entry factors (NIH3T3 4xM). Human CD81 was detected with the mouse monoclonal antibody 5A6 (Santa Cruz) and a secondary antibody coupled to APC. Mouse CD81 was detected with the monoclonal hamster antibody EAT2 coupled to PE (Santa Cruz). SR-BI expression was determined with a polyclonal rabbit serum (Novus Biologicals) reactive with both human and mouse SR-BI and a secondary antibody coupled to FITC. Grey histograms represent unstained cells. (B) Western Blot analysis of human or mouse CLDN1 and OCLN expressed in NIH3T3 cell derivatives. Both CLDN1- and OCLN-specific antibodies were from Zymed.
(1.30 MB EPS)

Figure S7 CD81 dependent infection of NIH3T3 cells stably expressing HCV entry factors of mouse or human origin with HCVpp. (A) NIH3T3 derived cell lines and Lunet N hCD81 cells were infected with concentrated MLV based pseudoparticles transducing Venus-GFP as reporter gene and carrying no envelope protein, the J6/wt glycoproteins, the J6/mCD81 glycoproteins, or as a control VSV-G. Cells were harvested 48 h after inoculation and the amount of infected cells was determined by flow cytometry and is depicted in the Dot Plots. (B) Neutralization of HCVpps with CD81 specific antibodies. Lunet N hCD81 cells were neutralized with the human CD81 specific antibody 5A6 and NIH3T3 4xM cells with the mouse CD81 specific antibody EAT1 (both Santa Cruz). Note that infections presented in (A) and (B) were performed in parallel. Control infections performed in the absence of CD81-specific antibodies are displayed in panel (A).
(3.05 MB EPS)

Figure S8 Characterization of HCV receptor expression in primary mouse hepatocytes. (A) Hepatocytes from C57BL/6 mice were isolated by a two-step collagenase perfusion method as described [66]. The expression of mouse CD81 and mouse SR-BI on the surface of primary mouse hepatocytes was determined by FACS analysis. CD81 was detected with the monoclonal hamster antibody EAT2 coupled to PE (Santa Cruz). SR-BI expression was determined with a polyclonal rabbit serum (Novus Biologicals) and a secondary antibody coupled to PE. Grey histograms represent unstained cells. (B) Western blot analysis of CLDN1 and OCLN expression in primary mouse hepatocytes. NIH3TE 4xM cells served as positive control. Both CLDN1- and OCLN-specific antibodies were from Zymed.
(1.76 MB EPS)
